# The Diagnostic Potential of Gut Microbiota-Derived Short-Chain Fatty Acids in Preeclampsia

**DOI:** 10.3389/fped.2022.878924

**Published:** 2022-06-03

**Authors:** Jialin Li, Lin Wang, Haimin Chen, Zhenglun Yang, Siqian Chen, Jiayi Wang, Yuping Zhou, Rongrong Xuan

**Affiliations:** ^1^Department of Obstetrics and Gynecology, The Affiliated Hospital of Medical School of Ningbo University, Ningbo, China; ^2^School of Medicine, Ningbo University, Ningbo, China; ^3^Ningbo Yinzhou No.2 Hospital, Ningbo, China; ^4^Key Laboratory of Applied Marine Biotechnology, Ministry of Education Ningbo University, Ningbo, China; ^5^Department of Gastroenterology, The Affiliated Hospital of Medical School of Ningbo University, Ningbo, China; ^6^Institute of Digestive Disease of Ningbo University, Ningbo, China

**Keywords:** preeclampsia, short-chain fatty acids, isobutyric acid, acetic acid, propionic acid, gut microbiota-derived metabolites

## Abstract

Preeclampsia (PE) is a complex pregnancy-related hypertensive disorder leading to multiorgan dysfunction. It has high maternal, fetal, and neonatal morbidity and mortality rates. The study of gut microbiota and its metabolites in PE deserves further exploration. Thirty-eight pregnant women with PE and 29 healthy pregnant women in the third trimester of their pregnancy were recruited in this study. We used a targeted metabolomics approach to evaluate the short-chain fatty acids (SCFAs) in serum samples. The correlation between SCFAs and clinical characteristics was also explored. The results of mass spectrometry (MS) showed significant differences at the metabolomics level of SCFAs between the PE and healthy control. The metabolic levels of acetate, propionate, isobutyrate, and valerate were significantly increased in the PE group than in the healthy control group. In contrast, caproic acid and butyrate levels were significantly reduced. The correlation analysis showed that urea, systolic, and diastolic blood pressure levels were positively correlated with four types of SCFAs (acetic acid, propionic acid, isobutyric acid, and valeric acid) which increased in the PE group. Furthermore, the neutrophil percentage and the fetal birth weight were negatively correlated with isobutyric acid and valeric acid. In addition, the receiver operating characteristic (ROC) analysis using a generalized linear model showed that multiple SCFAs would be potential diagnostic markers for PE, with high specificity, sensitivity, and area under the curve (AUC). Among them, isobutyric acid (sensitivity: 97.4%, specificity: 100%, AUC = 1.00), propionic acid (sensitivity: 86.8%, specificity: 93.3%, AUC = 0.954) and acetic acid (sensitivity: 86.8%, specificity: 83.3%, AUC = 0.891) depicted significantly higher diagnostic value and potential clinical applications. In summary, the results of this study indicate that SCFAs have the potential to become effective biomarkers for early screening of PE.

## Introduction

Preeclampsia (PE) is a pregnancy-specific, complex, and systemic disease. The global incidence of PE is 3–8%, and it is one of the leading causes of maternal, fetal, and neonatal morbidity and mortality in some cases (1, 2). The severity of PE varies significantly, and the state of an illness develops unpredictably in patients, further leading to maternal eclampsia, stroke, placental abruption, organ damage, and even death. In addition, PE can also lead to severe fetal and neonatal damage, including intrauterine growth restriction (IUGR), low birth weight (LBW) among infants, premature birth, stillbirth, and even neonatal death (3). PE is a multifactorial disorder with a complex pathogenesis involving maternal and placental vascular dysfunction. Potential abnormalities include incomplete spiral artery remodeling, endothelial damage, immune factors, imbalance of pro-and antiangiogenic substances, and various metabolic changes (4). Placental dysfunction is at the core of PE pathogenesis, but the specific mechanism has not been fully elucidated. Due to significant pathogenetic heterogeneity, no reliable biomarkers or clinical trials could predict the occurrence of PE during early pregnancy. Moreover, timely termination of pregnancy is the only effective treatment of PE to date, highlighting the significance of early diagnosis and treatment for improving the prognosis of PE (5). The International Federation of Gynecology and Obstetrics (FIGO) suggested that maternal risk factors, including maternal age, obesity, and family history along with biomarkers, could efficiently predict the risk of PE. Early clinical intervention among high-risk populations can significantly reduce the risk and severity of PE.

Therefore, from the point of view of gut microbiota-derived metabolites, we applied a targeted metabolomics approach to evaluate the SCFAs in serum samples of PE and healthy controls to help us understand the variations of SCFAs metabolism profiles. In addition, this study attempted to explore whether SCFAs could be used as biomarkers for diagnosing PE. Finally, we further analyzed SCFAs related metabolic pathways and mechanisms to provide early PE diagnosis and intervention references.

## Materials and Methods

### Participants

We recruited 38 pregnant women with PE and 29 healthy controls who delivered at the Affiliated Hospital of Medical School of Ningbo University from October 2020 to June 2021. The inclusion criteria were gestational week ≥ 28 weeks, normal blood pressure before pregnancy and voluntary participation. The exclusion criteria for the patients included those who suffered from gestational hypertension, took antibiotics, probiotics, and prebiotics within 1 month of sampling or suffered from diarrhea and other gastrointestinal symptoms.

The 2020 diagnostic criteria for preeclampsia provided by the American College of Obstetricians and Gynecologists (ACOG) was applied. Gestational hypertension is defined as the new-onset systolic and/or diastolic blood pressure ≥140/90 mmHg during double blood pressure measurements at least 4 h apart after 20 weeks of gestation. Blood pressure can return to normal during postpartum. Preeclampsia is characterized as 24-h urine protein > 300 mg or protein/creatinine ratio ≥ 0.3 mg/dl depending on gestational hypertension; urine protein ≥ 2+; or proteinuria absence with new onset of any of the following: ①thrombocytopenia: platelet count <100 × 10^9^/L; ②renal insufficiency: serum creatinine > 1.1 mg/dl or a doubling of the normal upper limit, in the absence of other kidney diseases; ③impaired liver function: transaminases are twice the average concentration; ④pulmonary edema; ⑤medication unresponsiveness to new-onset headache, and other causes or blurred vision were excluded. Throughout the pregnancy, pregnant women with normal blood pressure levels, no diabetes, cardiovascular and cerebrovascular diseases and metabolic diseases before pregnancy and no other complications of pregnancy were categorized under the normal pregnant (NP) group.

This study was approved by the Institutional Review Board (IRB) of the Affiliated Hospital of Medical School of Ningbo University (KY202011224). Furthermore, all the methods performed in this study adhered to the Declaration of Helsinki principles, and signed written informed consent was obtained from all participants.

### Collection of Clinical Data and Biological Samples

We included the clinical information of the two data groups, including age, vital signs, height, weight, pre-pregnancy BMI, gravidity, parity, and pregnancy outcomes. In addition, biochemical assays included triacylglycerol, total cholesterol, low-density lipoprotein cholesterol, high-density lipoprotein cholesterol, white blood cell count, hemoglobin, alanine aminotransferase, aspartate aminotransferase, and total bile acid, collected at the same time.

The cubital venous blood was collected from the participants after fasting for 8–10 h and further centrifuged (4°C, 4000 g, 7 min) to obtain serum after standing still for 1 h. The serum samples obtained were stored at−80°C until metabolomic analysis.

### Targeted Metabolomic Analysis of SCFAs

Serum samples underwent targeted metabolomic analysis using gas chromatography-mass spectrometry (GC-MS). First, an adequate serum sample (20 μL) mixed with 15% phosphoric acid (50 μL), 75μg/mL internal standard solution (isocaproic acid, 10 μL), and ether (140 μL) were pipetted precisely to pretreat, derive, and extract target analytes. Next, samples were centrifuged using 12,000 rpm at 4°C for 10 min, and the upper organic layer was collected for analysis using Thermo TRACE 1310-ISQ LT GC-MS (Thermo, US). The sample was injected in split mode (10:1), and helium (1 mL/min) was used as the carrier gas. SCFAs were undergone using an HP-INNOWAX column (30m × 0.25mm, 0.25μm; Agilent, US) with Electrospray ionization (ESI) source having positive ionization mode. The small molecules were measured using GC-MS. The temperatures of the chromatographic inlet, ion source, transfer line, and the quadrupole mass spectrometer were regulated at 250, 230, 250, and 150°C, respectively. The starting temperature of the programmed temperature rise was 90°C that increased to 120°C at 10°C/min, and then to 150°C at 5°C/min. Finally, the temperature was elevated to 250°C at 25°C/min for 2 min. At last, the obtained extracts were assayed for analytes using GC-MS.

### Metabolomics Data Analysis

The potential differential metabolites were analyzed using the principal component analysis (PCA), partial least squares discriminant analysis (PLS-DA), orthogonal partial least squares discriminant analysis (OPLS-DA), and linear regression. Quantitative analysis of serum metabolite was performed with R through multivariate statistical analyses (version 3.1.3, pheatmap and ropls package, function cor and cor. test) for calculating SCFAs concentration, data evaluation, and testing. Pathway analysis was performed using the Kyoto Encyclopedia of Genes and Genomes (KEGG; Kyoto, Japan). In addition, receiver operating characteristic curve (ROC) analysis was performed, and the area under the curve (AUC) assessed the predictive diagnostic power of the metabolites. Pearson's correlation coefficient or Spearman's rank correlation coefficient was used to determine the correlation among individual metabolites.

### Statistical Analysis

The statistical analysis was performed using the SPSS19.0 software. The two independent sample *t*-test assessed differences between groups, and the Wilcoxon rank-sum test was used for non-normally distributed data. Continuous variables were expressed as mean ± standard error (SD). Differences were compared using the chi-square test or the Fisher's exact test for categorical variables. A *p*-value < 0.05 was considered statistically significant.

## Results

### Baseline Data of PE Group and NP Group

Thirty-eight pregnant women with PE and 29 healthy pregnant women were included in this study. The baseline characteristics of the PE and NP groups are shown in Table 1, and the maternal prenatal blood parameters are depicted in Table 2. In the third trimester of pregnancy, both systolic and diastolic blood pressure were significantly increased in PE (*p* < 0.05). Compared with controls, pregnant women with PE had substantially higher total bile acid, urea, and urea/creatinine ratio. Moreover, they exhibited a decreasing trend for white blood cell count, neutrophil percentage, total cholesterol level, and thrombin time (*p* < 0.05). Based on pregnancy outcomes between the two groups, the gestational delivery age in the PE group was significantly earlier (*p* < 0.01), and the fetal birth weight was significantly lower (*p* < 0.01). In addition, the cesarean section rate in the PE group (71%) was significantly higher than that in the control group (*p* < 0.05). Other variables like age, pre-pregnancy BMI, weight gain during pregnancy, parity, hemoglobin, total, direct, indirect bilirubin, albumin, and platelet counts did not reveal any significant difference between the two groups (*p* > 0.05).

**Table 1:** General characteristics of the PE and NP groups.

**Variables**	**NP** **(*n* = 29)**	**PE** **(*n* = 38)**	***p*-value**
Systolic blood pressure (mmHg)	120.10 ± 9.58	147.42 ± 15.70	<0.001
Diastolic blood pressure (mmHg)	74.07 ± 6.76	94.39 ± 11.16	<0.001
Age (yrs)	28.19 ± 2.39	29.82 ± 4.78	0.089
Height (cm)	163.14 ± 5.11	161.16 ± 5.04	0.118
Prenatal BMI (kg/m2)	22.02 ± 2.90	23.08 ± 3.83	0.294
Weight gain (kg)	14.35 ± 4.12	14.54 ± 3.84	0.843
Gravidity	1.97 ± 1.18	2.03 ± 1.37	0.854
Parity, *n* (%)			0.407
Primipara	17 (0.59)	26 (0.71)	
Multiparous	12 (0.41)	12 (0.29)	
Gestational age (weeks)	39.43 ± 0.83	37.80 ± 1.98	<0.001
Fetal birth weight (kg)	3.46 ± 0.43	2.90 ± 0.64	<0.001
Delivery mode, *n* (%)			<0.001
Vaginal delivery	23 (0.79)	11 (0.29)	
Cesarean delivery	6 (0.21)	27 (0.71)	

**Table 2:** Clinical characteristics of maternal blood in PE and NP groups.

**Variables**	**NP** **(*n* = 29)**	**PE** **(*n* = 38)**	***p*-value**
**Blood biochemical indicators**			
Total bilirubin (μmol/L)	8.78 ± 2.91	7.96 ± 3.32	0.126
Direct bilirubin (μmol/L)	1.47 ± 0.73	1.39 ± 0.71	0.309
Indirect bilirubin (μmol/L)	1.47 ± 0.73	6.57 ± 2.97	0.300
Triglycerides (mmol/L)	3.65 ± 0.88	4.27 ± 1.45	0.260
Total cholesterol (mmol/L)	6.56 ± 1.47	5.55 ± 0.89	0.028
Low density lipoprotein (mmol/L)	3.53 ± 1.00	3.04 ± 0.64	0.122
Alanine aminotransferase (U/L)	9.48 ± 3.21	12.00 ± 8.28	0.218
Aspartate aminotransferase (U/L)	19.69 ± 3.52	20.79 ± 8.67	0.914
Albumin (g/L)	37.85 ± 6.40	35.24 ± 3.63	0.071
Total bile acids (μmol/L)	2.69 ± 1.35	4.46 ± 2.59	<0.001
Urea (mmol/L)	3.26 ± 0.75	4.02 ± 1.02	<0.001
Serum creatinine (μmol/L)	46.63 ± 6.07	52.50 ± 12.58	0.154
Urea/creatinine ratio	0.07 ± 0.02	0.08 ± 0.02	0.045
Serum uric acid (μmol/L)	326.90 ± 75.77	368.03 ± 96.42	0.050
High-density lipoprotein (mmol/L)	2.01 ± 0.36	1.73 ± 0.37	0.081
**Blood routine index**			
Platelets (*10^∧^9/L)	191.07 ± 46.64	191.95 ± 52.87	0.894
White blood cells (*10^∧^9/L)	10.83 ± 3.21	8.81 ± 2.28	0.002
Hemoglobin (g/L)	127.21 ± 12.33	125.89 ± 12.11	0.664
Neutrophil percentage (%)	78.41 ± 6.31	74.03 ± 6.50	0.007
C reactive protein	5.47 ± 5.68	4.12 ± 2.67	0.711
**Coagulation function index**			
Plasma prothrombin time PT (s)	11.06 ± 0.59	11.03 ± 0.42	0.827
Activated partial thromboplastin time APTT (s)	26.90 ± 1.57	26.56 ± 1.81	0.425
Thrombin time TT (s)	16.66 ± 1.39	15.59 ± 1.90	0.043
Plasma fibrinogen determination (g/L)	3.77 ± 0.62	3.71 ± 0.93	0.784
D-dimer (μg/L)	603.83 ± 339.51	534.22 ± 364.06	0.526
Determination of fibrin (ogen) degradation products (μg/ml)	7.50 ± 3.67	6.63 ± 3.64	0.355

### Serum SCFAs Metabolism Profiles Were Significantly Different Between PE Group and NP Groups

**Figure 1:**
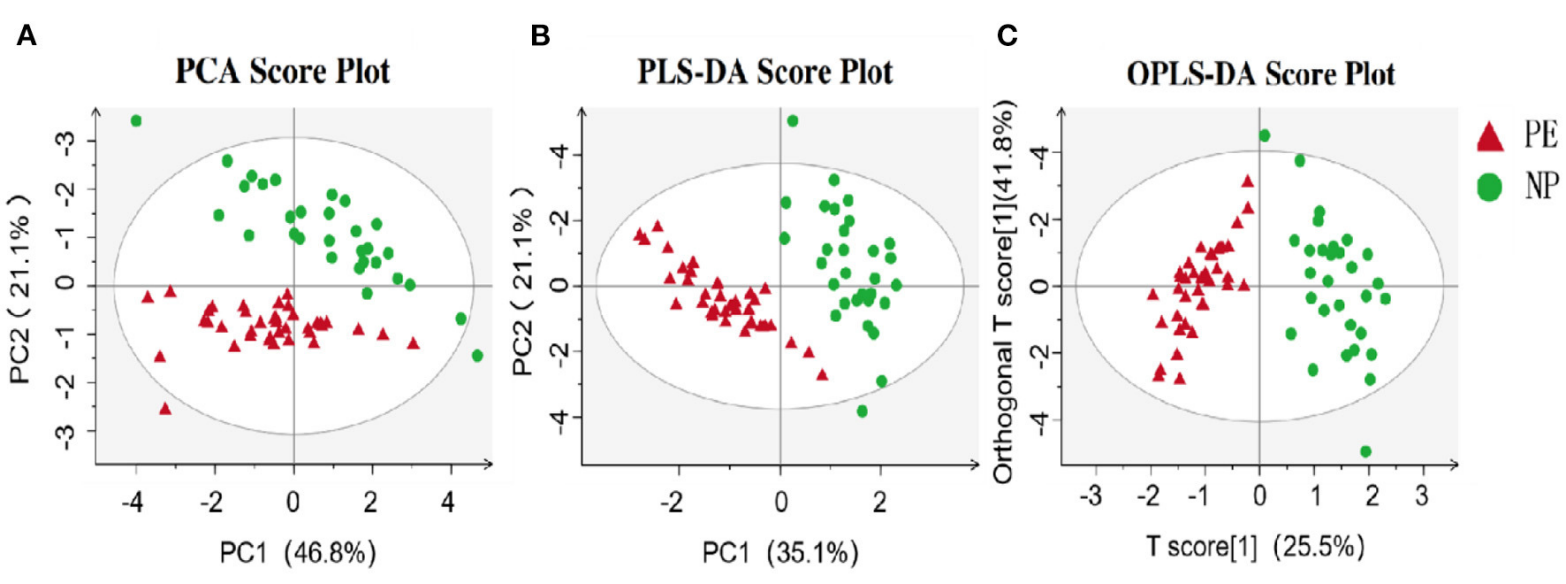
Metabolomics analysis of PE and NP groups. **(A)** PCA; **(B)** PLS-DA; **(C)** OPLS-DA. The abscissa is the first principal component (PC1), the ordinate is the second principal component (PC2), and the numbers in parentheses represent the proportion of the corresponding principal components in the comprehensive original information. One point in the figure corresponds to one sample, green represents the NP group, and red represents the PE group.

In this study, PCA was employed for unsupervised analysis and PLS-DA for supervised pattern recognition, overviewing data, and detecting SCFAs trends in the experiments. As shown in the PCA score plot (Figure 1A), the PE group had an evident separation trend from the control group, indicating a specific difference in the SCFAs between the two groups. The supervised multivariate statistical analysis methods, including PLS-DA and OPLS-DA, were used to remodel and analyze the data to verify further the statistical differences between the two groups of samples. The results are shown in Figures 1B,C. Again, these were separated and aggregated well within the same group, indicating specific differences in the SCFAs between the two groups.

**Figure 2:**
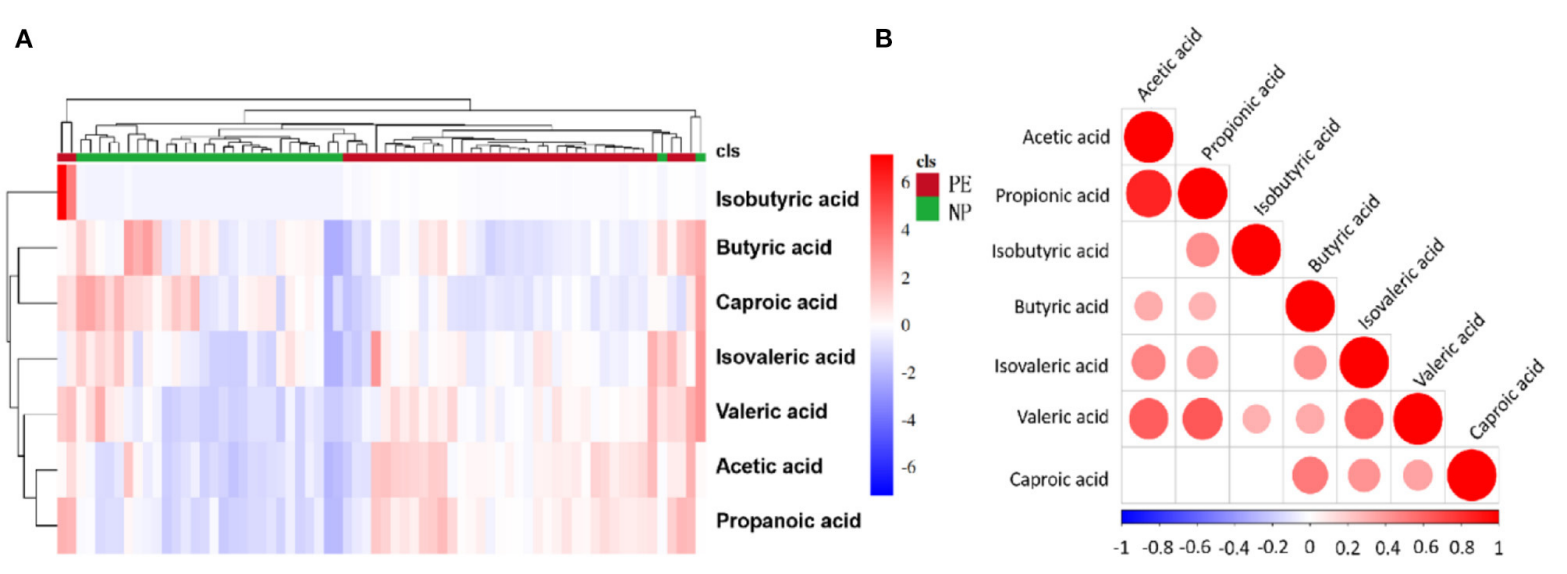
Heatmap and correlation heatmap of metabolites between PE and NP groups. The magnitude of the relative amounts in **(A,B)** is shown by the difference in color, where the columns represent samples, and the rows represent metabolites. Different colors represent different intensities, with red indicating relatively high kurtosis values and blue indicating relatively low kurtosis values.

The relationship between samples was more intuitively and comprehensively evaluated to reveal the differences between the expression patterns of SCFAs in different samples. For this, agglomerative hierarchical clustering analysis was performed on each group to accurately screen marker metabolites and explore changes within corresponding metabolic processes. A metabolite heatmap was generated between the PE and NP groups (Figure 2A), showing the levels of acetic acid, propionic acid, isobutyric acid, and valeric acid were significantly increased in the PE group. The elevated levels hinted that these SCFAs could differentiate between the two groups. Furthermore, the heat map revealed a positive correlation between valeric acid and the remaining six types of SCFAs, with the strongest correlation between acetate and propionate (Figure 2B).

### Quantitative Analysis of Serum SCFAs in PE Group and NP Group

**Figure 3:**
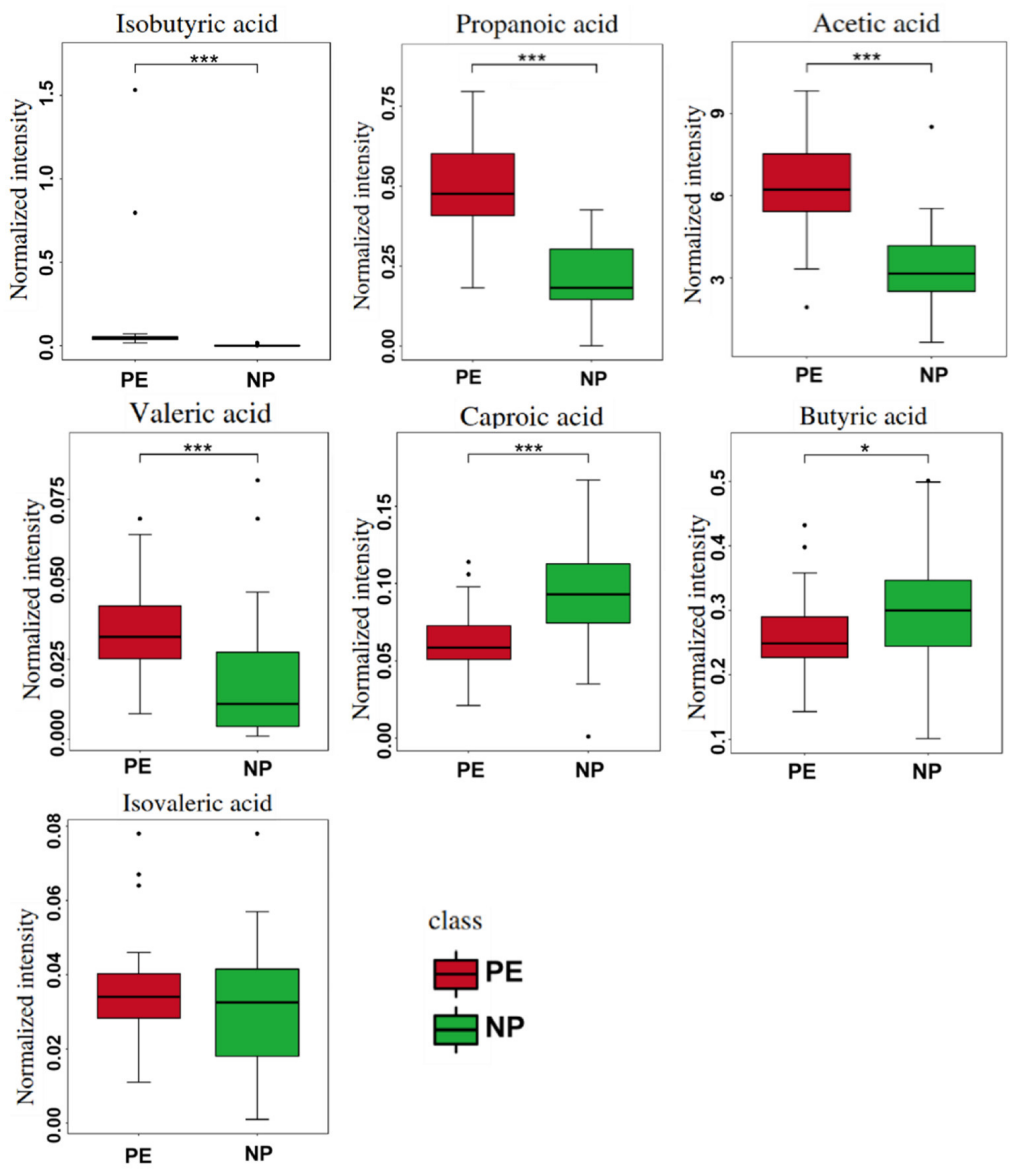
Quantitative comparison of serum short-chain fatty acids (SCFAs) between PE and NP groups. *P < 0.05, ***P < 0.001.

We analyzed the metabolic levels of seven SCFAs between the PE and NP groups. The results are shown in Figure 3. Compared with the NP group, the PE group had significantly higher levels of acetate, propionate, isobutyrate, and valerate (*p* < 0.05). Acetate had the highest level, followed by propionate and butyrate. The change in isobutyric acid was the most significant, 42.450 times than that in the NP group.

### SCFAs Have High Sensitivity and Specificity in the Diagnosis of PE

**Figure 4:**
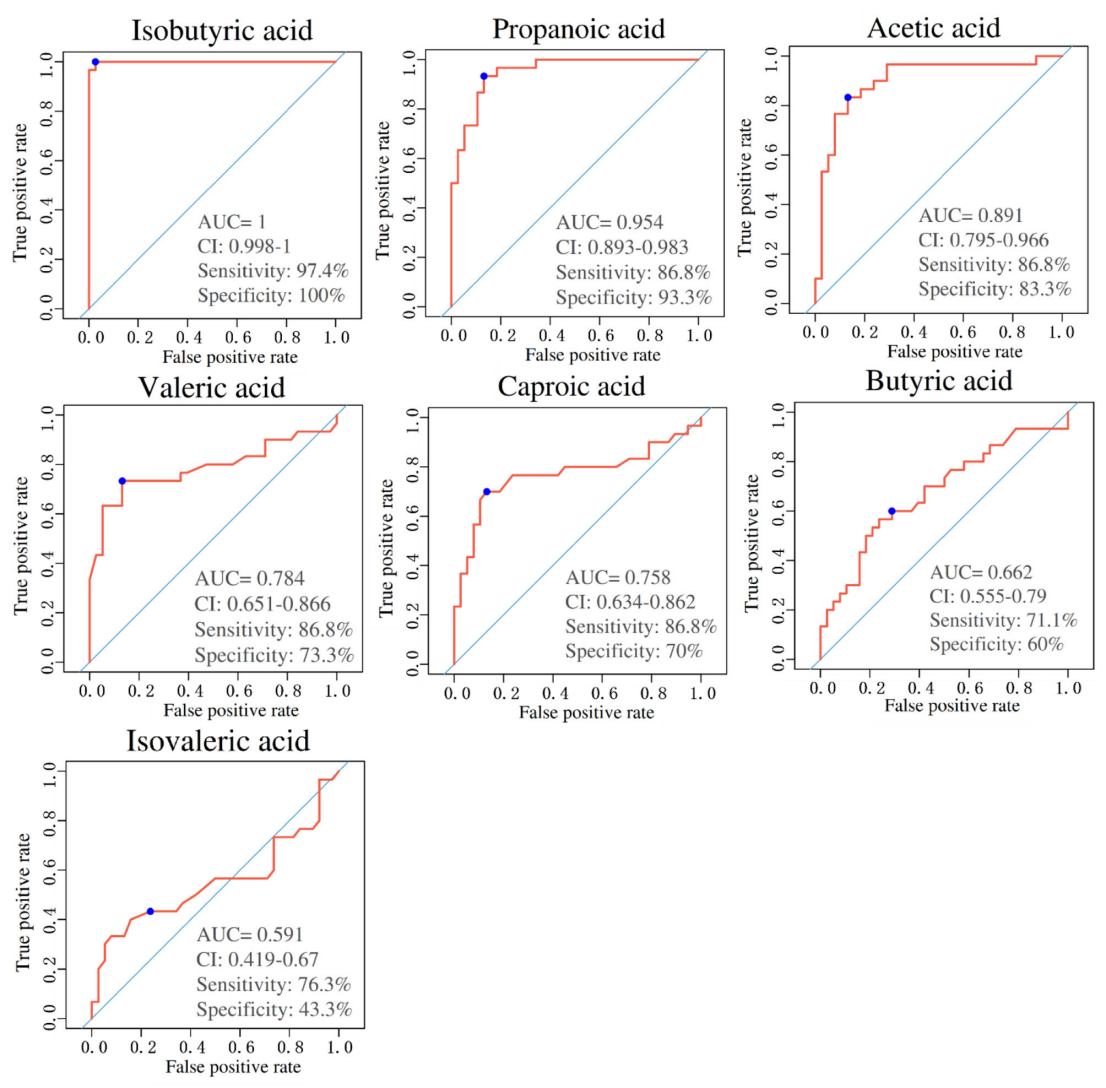
Area under the curves of short-chain fatty acids (SCFAs) between PE and NP groups. ROC curves for different metabolite combinations. AUC, Area under the curve. AUC > 0.5, indicating that it has high predictive ability.

ROC curve analysis was undergone on seven SCFAs levels between the PE and NP groups to evaluate their diagnostic marker potential (shown in Figure 4). The results depicted that isobutyric acid (sensitivity: 97.4%, specificity: 100%, AUC = 1.00), propionic acid (sensitivity: 86.8%, specificity: 93.3%, AUC = 0.954), acetic acid (sensitivity: 86.8%, specificity: 83.3%, AUC = 0.891) and valeric acid (sensitivity: 86.8%, specificity: 73.3%, AUC = 0.784) could become serum biomarkers to diagnose PE, and demonstrate good clinical application potential.

### Correlations Between Differential Clinical Indicators and Levels of SCFAs

**Figure 5:**
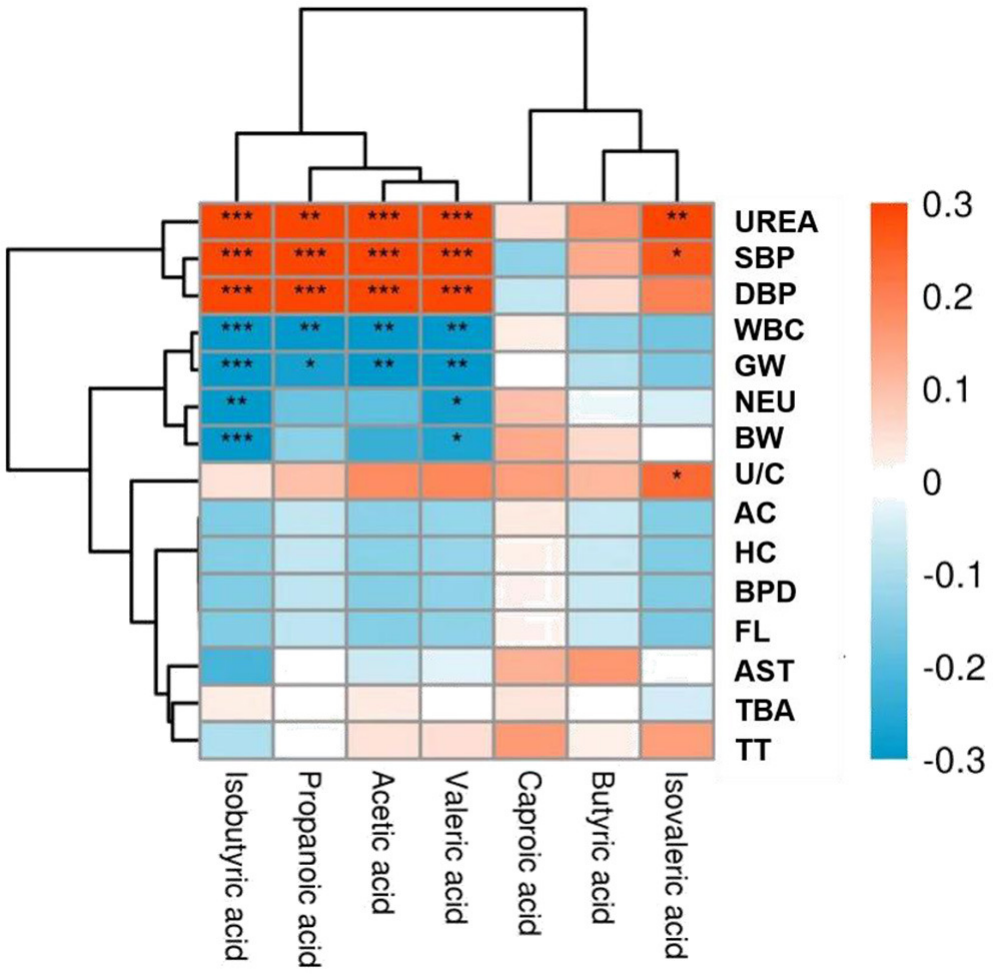
Heat map of correlations between short-chain fatty acids (SCFAs) and clinical indicators. Red and blue squares represent positive and negative correlations, respectively. Statistical significance is indicated on the square (*P < 0.05; **P < 0.01; ***P < 0.001). UREA, Urea; SBP, Systolic blood pressure; DBP, Diastolic blood pressure; WBC, White blood cells; GW, Gestational week; NEU, Neutrophil percentage; BW, Birth weight; U/C, Urea/creatinine ratio; AC, Abdominal circumference; HC, Head circumference; BPD, Biparietal diameter; FL, Femur length; AST, Aspartate aminotransferase; TBA, Total bile acid; TT, Thrombin time.

Correlation analysis was performed to explore further potential correlations of key clinical indexes with SCFAs in PE. As shown in Figure 5, both systolic and diastolic blood pressure showed substantial positive correlations with acetic acid, propionic acid, isobutyric acid, and valeric acid (*p* <0.001), consistent with the quantitative analysis results. In addition, isovaleric acid was also positively correlated with systolic blood pressure (*p* < 0.05) (6). However, no significant difference was observed in the isovaleric acid level between the two groups in this study. Apart from blood pressure, urea level, a risk factor for hypertension, was also positively correlated with the above SCFAs. These findings suggest the potential predictive value of SCFAs in PE. Furthermore, SCFAs were negatively correlated with the gestational week (GW) and fetal birth weight (BW). Many pertinent studies have found that an increase in maternal blood pressure was negatively associated with gestational age at delivery and offspring birth weight (7, 8). However, inflammatory blood markers, including white blood cell count (WBC) and neutrophil (NEU) percentage, were negatively correlated with SCFAs. The findings were consistent with the study by Shen et al. (9), which demonstrated that SCFAs have anti-inflammatory effects. Therefore, these findings suggest SCFAs are significantly associated with maternal clinical features and fetal pregnancy outcomes.

### Metabolic Pathway Analysis

**Figure 6:**
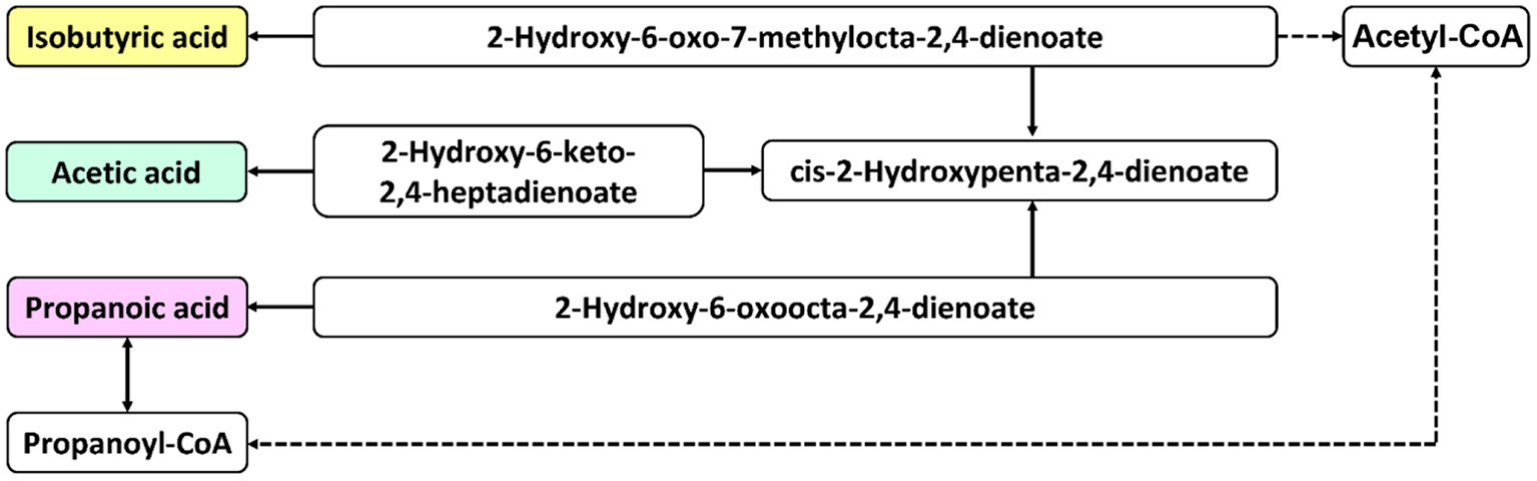
Schematic diagram of metabolic pathways. The solid arrows indicate direct effects, and the dashed arrows indicate indirect effects.

The differential metabolites were subjected to correlation analysis and KEGG pathway prediction. The predicted upstream and downstream relationships of SCFAs are shown in Figure 6. Acetate is the main component of SCFAs, and several gut microbiotas metabolize pyruvate using the acetyl-CoA or the Wood-Ljungdahl pathway. First, propionate is formed by converting succinate to methylmalonyl-CoA by the succinate pathway. Then, two acetyl-CoA molecules are condensed and reduced to butyryl-CoA, thereby converting to butyrate by phosphobutyryltransferase and butyrate kinase. In addition, butyryl-CoA can also be converted to butyrate through the butyryl-CoA: acetate CoA-transferase pathway. These substances are primarily related to xenobiotic biodegradation and lipid, amino acid, and glucose metabolism.

## Discussion

The gut microbiota plays an important role in host metabolism, immunity, and nutrition absorption. Furthermore, imbalance in the gut microbiota composition is linked to host metabolic abnormalities and systemic inflammation, which contributes to the development of many diseases, such as obesity, type 2 diabetes, atherosclerosis, non-alcoholic fatty liver disease, hypertension, and chronic kidney diseases (10). So far, limited research has been done on the impact of gut microbiota composition on PE. The present study showed that systolic blood pressure (SBP) is negatively correlated with the abundance of the butyrate producers Odoribacter and Clostridiaceae (11). A previous study reported that at late gestation in the PE patients, there was an overall increase in abundance of Clostridium perfringens and Bulleidia moorei but a reduction in Coprococcus catus (11). Another study reported that Firmicutes showed a decreased abundance in PE patients. What's more, Firmicutes produces several types of short chain fatty acids (SCFA) which may affect blood pressure and renal function (12).

The gut microbiota can produce a variety of metabolites, such as short-chain fatty acids (SCFA), trimethylamine/trimethylamine oxide, bile acids, lipopolysaccharide, and peptidoglycan, among which SCFAs are the final significant metabolites produced by the gut microbiota and have fundamental roles in maintaining gut homeostasis and regulating energy metabolism. SCFAs can ward off pathogens, regulate metabolism, maintain glucose and fat homeostasis, interact with endocrine and immune systems, and affect drug metabolism and absorption (12–14). The most abundant gut SCFAs are acetic, propionic, and butyric acids, with relatively low isobutyric, valeric, and isovaleric acid levels. The SCFAs content depends on various factors, including diet, age, genotype, of which the gut microbiota has the most significant impact (15, 16). According to reports, *Firmicutes* were reduced in PE patients, and the SCFAs produced by *Firmicutes* could affect blood pressure and renal function (12). Studies have found that changes inside the gut microbiota impact blood pressure regulation (13, 17). There is also a specific correlation between gut microbiota-derived metabolites, such as SCFAs, and blood pressure (18). Two clinical studies revealed significantly lower levels of microbial diversity and SCFAs-producing bacteria in patients with hypertension and PE compared with healthy pregnant women (12). However, there are only a few studies on the changes of SCFAs in PE patients, which requires further exploration.

We found significant differences in the levels of SCFAs between the PE group and the NP group in this study. The levels of acetic acid, propionic acid, isobutyric acid, and valeric acid in the serum of PE patients were significantly elevated, while butyric acid and caproic acid were reduced considerably. However, Chang et al. (17) found that the expression of valeric acid in the PE group was significantly lower than that in the healthy pregnancy control group, which was inconsistent with our findings. The heterogeneity in subjects, regions and their diets could explain this difference. For instance, evidence indicates that diet is one of the most important factors regulating the gut microbiota and can rapidly modify microflora composition through direct and indirect mechanisms within a few days (15). Moreover, SCFAs changes were significantly correlated with maternal blood parameters and fetal pregnancy outcomes. SCFAs were found to be positively correlated with blood pressure and urea. However, they were negatively associated with inflammatory markers of maternal blood (WBC and NEU percentage), GW at delivery, and fetal BW. The implications of SCFAs and metabolic disorders during pregnancy are critical to maternal health and fetal development. ROC curve analysis showed that isobutyric acid (highest fold difference), propionic acid (high absolute expression with more than two times fold), and acetate (highest absolute expression) could be potential PE biomarkers with significantly high sensitivity and specificity. It has the potential to serve as a novel biomarker for clinically diagnosing and monitoring PE.

Acetate, the most abundant SCFAs in the gut, is metabolized by various gut microbes, including Bifidobacterium (19, 20). Previous studies had confirmed that SCFAs were essential mediators between the microbiota and blood pressure regulation in the host (21). The study also described that the olfactory receptor Olfr78 and the GPCR Gpr41 were expressed in the smooth muscle cells of small resistance vessels. Therefore, these act as receptors of SCFAs and mediate renin secretion to regulate blood pressure. Moreover, studies have demonstrated that SCFAs receptors significantly regulate the two critical determinants of systemic blood pressure (renin secretion and vascular tone) and are controlled by signals generated by gut microbes (22). A study revealed that acetic acid, propionic acid, and isobutyric acid levels were significantly increased in a hypertensive rat model (23). This study showed that being the highest expression SCFAs, acetate had a significant difference between the PE and control groups. Isobutyric acid is a kind of SCFA with four carbon atoms, a branched single-chain fatty acid produced by the gut microbiota. It could be a marker of *Clostridium bifidum* colonization (16). However, isovaleric acid is present at low levels in the gut, with little research confirming its potential. This study showed that isobutyric acid was more than 40-fold elevated in PE patients than healthy controls. Previous studies have shown that the concentration of isobutyric acid is positively correlated with *Rikenbacteriaceae* and *Rumenococcus* (24), while *Rumenococcus* is significantly enriched in the fecal samples of PE patients (12, 25). These findings suggest that *Ruminococcus* enrichment could substantially increase isobutyric acid.

Propionic acid is the primary metabolite of Bacteroidetes fermentation, inhibiting cholesterol synthesis (16). Based on previous studies, the clinical effects of propionic acid on lipid metabolism are manifested through lowering cholesterol concentrations and reducing fat storage (26). The present study found that propionic acid levels were significantly increased in PE patients. In addition, total serum cholesterol was considerably lower in PE patients than healthy pregnant women, confirming the promoting effect of propionic acid on lipid metabolism. Previous studies suggest that hypertension is correlated with a decrease in SCFAs production, directly regulating blood pressure by binding to receptors. The newly discovered SCFAs receptors are mainly GPCRs (27). Jennifer et al. (21) confirmed that Gpr41 and Olfr78 were propionate receptors (22). It is worth mentioning that SCFAs binding to different receptors plays diametrically opposite roles in blood pressure regulation (27). Gpr41 receptor expressed in vascular smooth muscle cells and endothelial cells is involved in the hypotensive effect on propionic acid through vasodilation. Moreover, this SCFA receptor produces an acute hypotensive response in wild-type mice by inducing *in vitro* expansion (22). On the other hand, activation of Olfr78 receptors distributed in the kidneys and blood vessels can increase blood pressure and antagonize the hypotensive effect of propionic acid, and its expression may affect baseline blood pressure (22, 27). This study found that the propionic acid level of PE patients was significantly higher than that of the NP group, and their blood pressure levels were also significantly increased, which may be related to the boosting effect of propionic acid O1fr78 receptor binding.

In addition, Gomez-Arango et al. (28) conducted another clinical study on hypertensive disorders during pregnancy (including gestational hypertension, preeclampsia, and HELLP syndrome). They reported that butyrate production was inversely associated with blood pressure. Our study found that the butyrate level of patients with PE was significantly lower than that in normal pregnant women. At the same time, the blood pressure was considerably higher, consistent with the results from previous studies.

In general, this study utilized a targeted metabolomics approach to analyze the metabolic profiles of SCFAs from the serum of PE patients and normal pregnant women. Our study found the expression changes and clinical significance of serum SFCAs in patients with PE. Several SFCAs were screened as potential biomarkers for the early diagnosis of PE. However, some limitations of this study should be reiterated. First, the sample size was not significantly large, which could impact the consistency of the statistical analysis. In addition, certain external factors like dietary habits, comorbidities during pregnancy, and previous medications, could affect the metabolome. Second, the gut microbiota and gut microbiota-derived metabolites were not detected in the feces of PE patients. Therefore, the gut microbiota source of these metabolites remains unclear. Third, a large-scale clinical sample validation and further molecular mechanism studies were not performed. Lastly, the causal relationship between short chain fatty acids and preclampsia has not been studied. These limitations provide the follow-up research contents and future directions of the research group.

## Data Availability Statement

The original contributions presented in the study are included in the article/supplementary material, further inquiries can be directed to the corresponding authors.

## Ethics Statement

The studies involving human participants were reviewed and approved by Institutional Review Board (IRB) of the Affiliated Hospital of Medical School of Ningbo University. The patients/participants provided their written informed consent to participate in this study.

## Author Contributions

YZ and RX developed the concept and revised the manuscript. JL, LW, HC, ZY, SC, and JW conducted the experiments and the statistical analysis. JL and LW organized the structure and wrote the manuscript. All authors approved the final version.

## Funding

This project was funded by Ningbo Natural Science Foundation Project (No. 2021J239) and Ningbo Public Welfare Technology Plan Project (No. 2021S101).

## Conflict of Interest

The authors declare that the research was conducted in the absence of any commercial or financial relationships that could be construed as a potential conflict of interest.

## Publisher's Note

All claims expressed in this article are solely those of the authors and do not necessarily represent those of their affiliated organizations, or those of the publisher, the editors and the reviewers. Any product that may be evaluated in this article, or claim that may be made by its manufacturer, is not guaranteed or endorsed by the publisher.
